# Enhanced Immunogenicity of Adjuvanted Microparticulate HPV16 Vaccines Administered via the Transdermal Route

**DOI:** 10.3390/ph15091128

**Published:** 2022-09-09

**Authors:** Trinh Phuong Vo, Gitika Panicker, Kimberly Braz-Gomes, Ashwin C. Parenky, Ira Rajbhandari, Mangalathu S. Rajeevan, Elizabeth R. Unger, Martin J. D’Souza, Mohammad N. Uddin

**Affiliations:** 1 College of Pharmacy, Mercer University, Atlanta, GA 30341, USA; 2 Division of High-Consequence Pathogens and Pathology, National Center for Emerging and Zoonotic Infectious Diseases, Centers for Disease Control and Prevention, Atlanta, GA 30329, USA

**Keywords:** human papillomavirus (HPV), virus-like particle (VLP), microparticles, particulate vaccines, transdermal, adjuvant, antibodies

## Abstract

Human papillomavirus (HPV) causes cervical cancer among women and is associated with other anogenital cancers in men and women. Prophylactic particulate vaccines that are affordable, self-administered and efficacious could improve uptake of HPV vaccines world-wide. The goal of this research is to develop a microparticulate HPV16 vaccine for transdermal administration using AdminPatch^®^ and assess its immunogenicity in a pre-clinical mouse model. HPV16 microparticles were prepared using a biocompatible polymer and characterized in terms of size, zeta potential, encapsulation efficiency and microparticle yield. Scanning and transmission electron microscopy were conducted to confirm particle image and to visualize the conformation of HPV16 vaccine particles released from microparticle formulation. In vivo studies performed to evaluate the potential of the microparticulate vaccine initiated a robust and sustained immune response. HPV16 IgG antibodies were significantly elevated in the microparticle group compared to antigen solutions administered by the transdermal route. Results show significant expansion of CD4+, CD45R, CD27 and CD62L cell populations in the vaccinated mice group, indicating the high efficacy of the microparticulate vaccine when administered via transdermal route. The findings of this study call attention to the use of minimally invasive, pain-free routes to deliver vaccine.

## 1. Introduction

Papillomaviruses are a type of non-enveloped, double-stranded DNA viruses that infect mucosal and cutaneous epithelia in humans and other species [1,2]. Human papillomavirus (HPV) is a common sexually transmitted virus around the world [3]. Over 200 HPV types have been identified, of which about 40 affect the genital tract and are further subdivided by their potential to cause cancer. High-risk types cause cervical cancer and are often associated with vaginal, anal, penile, vulvar and oropharyngeal cancers [4]. According to the World Health Organization (WHO), cervical cancer is responsible for close to 300,000 deaths world-wide, ranking it the fourth most common form of cancer among women, with about 85% of the infections occurring in developing countries [5]. Currently, there are three prophylactic vaccines against HPV: Cevarix^®^ from Glaxo Smith Kline (Rixensart, Belgium), and Gardasil^®^ and Gardasil 9^®^ from Merck &Co. Inc. (Kenilworth, NJ, USA). Cervarix^®^ protects against HPV16 and 18, which cause 70% of cervical cancers. Gardasil^®^ protects against HPV16 and 18, along with HPV6 and 11, which cause genital warts. Gardasil-9^®^ contains antigens of additional five high-risk types, HPV 31, 33, 45, 52 and 58, and can protect against 90% of cervical cancers [4]. Although commercial vaccines are very efficacious, their high manufacturing cost, invasive administration, vaccine stability and need for cold-chain storage impose a significant financial burden on resource-limited regions of the world [6]. In addition, pain, needle injuries and poor patient compliance associated with vaccines administered via conventional injections, intramuscular and subcutaneous, are barriers to vaccine uptake world-wide [7]. Therefore, different immunization routes (oral, transdermal, intranasal) and alternative vaccine formulations have been investigated to increase vaccination uptake [8,9].

**Figure 1:**
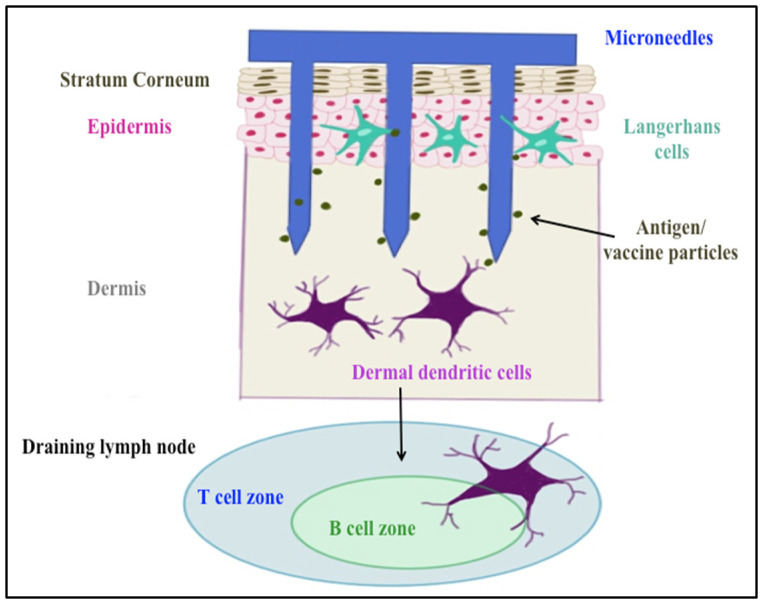
Skin layers and microneedle immunization targets.

Delivering vaccines via the transdermal route overcomes some of these hurdles. It is a promising immunization route, since approximately 20 billion immune cells of various subtypes such as Langerhans and dermal dendritic cells, as well as subsets of T-lymphocytes, reside under the skin [9]. Transdermal administration of particulate vaccines can induce both mucosal and systemic immune responses, whereas the conventional injections trigger mainly systemic immunity [10]. Microneedles such as AdminPatch^®^ perforate stratum corneum, the outer layer of the skin, allowing vaccines and other large molecules to reach antigen-presenting cells (APCs) in the epidermis and dermis layers (shown in Figure 1). In addition, microneedles avoid contact with nerve endings in the dermis because of their short needle length, resulting in painless vaccination [7]. This mode of delivery has previously been successful in eliciting a neutralizing antibody response to HPV antigens [11,12]. Corbett et al. used dry micro-projection arrays (Nanopatch^TM^) containing vaccine material (Gardasil^®^) to deliver to C57BL/6 mouse ear skin, resulting in the production of peak neutralization antibodies against HPV at day 28 that were sustained up to 16 weeks post immunization [11]. Kines et al. designed a vaccine formulation with HPV pseudovirus-encapsidated plasmids targeting skin using microneedles. The results showed microneedle delivery was similarly effective as intramuscular delivery in terms of inducing a neutralizing antibody (nAb) response against HPV16 at three weeks from the last immunization [12].

Use of particulate vaccines also offers advantages over conventional vaccines that are administered solution form. Particulate vaccines have been proven to be more immunogenic than soluble antigens, since the particulate forms are better recognized as “more foreign (more antigenic)” by the system, resulting in increasing vaccine uptake by the APCs via the phagocytosis pathway [13]. In this signaling process, the APCs present the antigens on their surface that initiate adaptive immune responses by communicating with T cells and B cells of spleen and lymph nodes [13]. Encapsulation of vaccines in micro/nanoparticles enhances antigen stability and prolongs antigen exposure in the body; consequently, a lower amount of antigen is required for dosing [14].

Adjuvant is another important component of vaccines. Most commercial vaccines contain adjuvants, which are substances that play several important roles such as the recruiting of APCs to the injection site, enhancing antigen uptake, prolonging antigen exposure time and sustaining long-term protection against diseases [15]. For instance, Gardasil^®^ contains amorphous aluminum hydroxyphosphate sulfate to adsorb HPV6, 11, 16 and 18 VLPs (virus-like particle), increasing antigen stability and recruiting APCs to the injection site [16]. The Cervarix^®^ vaccine contains AS04 adjuvant, which contains aluminum hydroxide and monophosphorylate lipid A (MPL), the first immunostimulatory Toll-like receptor agonist (TLR) 4 to be used as a vaccine adjuvant in humans. The presence of AS04 adjuvants was proven to elevate innate responses, enhance adaptive immunity, elicit higher antibody titers after the first dose and improve memory B cells up to 2 years post vaccination [17]. Cholera toxin (CT) is a potential mucosal adjuvant, which has been evaluated in vaccines via oral and intranasal routes [18,19]. The biological function of CT subunit B is to bind to the GM1 ganglioside receptor on the intestinal cells and enhance the antigen engulfment when administered orally. Even though how CT elicits the immune response is not fully understood, researchers have used it in vaccine formulations as a promising adjuvant [18].

Our approach concerns the use of adjuvanted microparticulate vaccines delivered transdermally in a pre-clinical mouse model. The goal of the study was to investigate two microparticulate (MP) formulations with combinations of adjuvants: CT + MPL and alum + MPL, along with HPV16 VLP as an antigen. MPs were characterized and evaluated for their efficacy in BALB/c mice.

## 2. Results

### 2.1. Characterization of Microparticles

**Figure 2:**
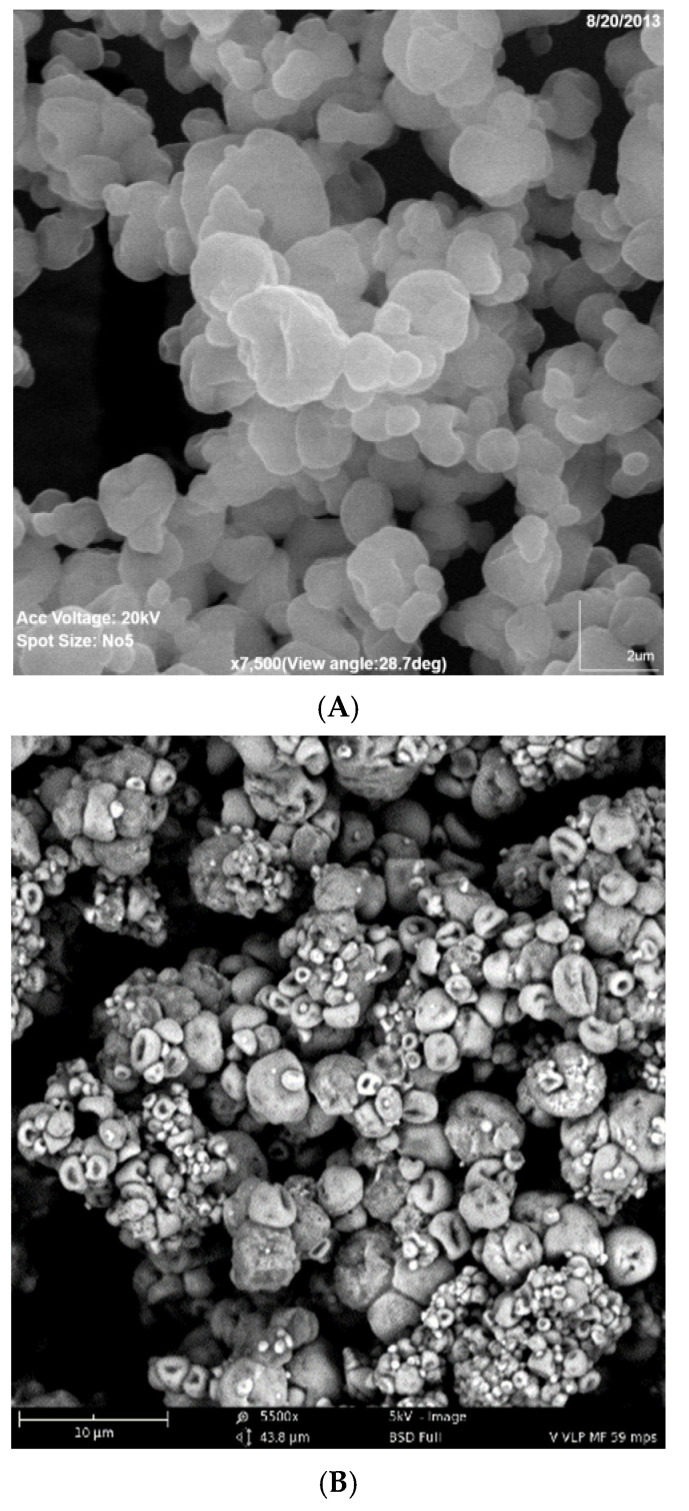
(**A**,**B**). Scanning Electron Microscope Image of Formulations 1 and 2. (**A**) Formulation 1 (HPV16VLP MPs). (**B**) Formulation 2 (HPV16VLP-MPL-alum-MPs).

**Figure 3:**
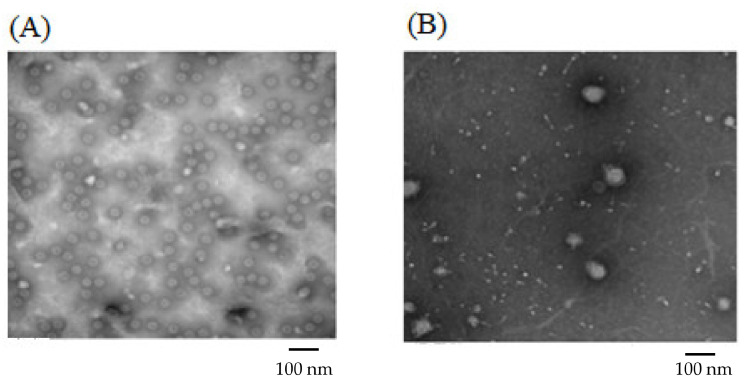
Transmission Electron Microscopy Images of Formulation 1 and Formulation 2. (**A**): Formulation 1 contained 60% CPD and HPV16 VLP alone (prior to mixing with adjuvant MPs). (**B**): Formulation 2 contained 20% CPD, 5% vaccine and adjuvant loading HPV16 VLP, MPL and alum.

**Figure 4:**
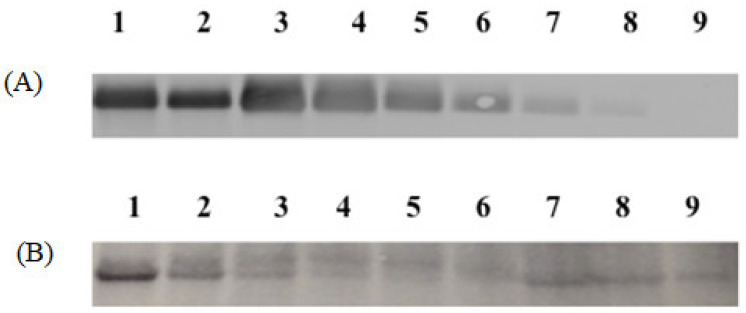
Western blot images of Formulations 1 and 2. (**A**): Formulation 1. Lane 1, 2: HPV16 VLPs in MPs (1.0 and 0.5 μg), Lane 3, 4, 5, 6, 7 and 8: blank MPs + HPV 16 VLPs in solution (1.0, 0.5, 0.25, 0.125, 0.06 and 0.03 μg), Lane 9: Blank MP. (**B**): Formulation 2 contained 20% CPD, and HPV16 VLP absorbed into alum and MPL. Lane 1, 2, 3, 4 and 5: VLPs + Blank MPs (2.0, 1.0, 0.5, 0.25 and 0.125 μg), Lane 6: Blank MP, Lane 7, 8 and 9: HPV16 VLPs + Alum + MPL MPs (2.0, 1.0 and 0.5 μg).

For Formulation 1, the percentage yield of antigen MPs was 91% ± 3.4% (*w*/*w*) and encapsulation yield was 80.5 ± 0.85%. The particle size ranged from 1–5 μm in diameter with an average size of 3.45 ± 0.41 μm and the charges were −20.54 ± 0.52 mV. SEMs of MPs are shown in Figure 2A, which also confirms that the particles are smaller than 5 μm. TEM imagery shown in Figure 3A proves that the conformational structure of HPV16 VLPs was still intact after release from encapsulation. HPV16 L1 protein released from microparticles was detected by Western blot to calculate encapsulation yield (Figure 4A).

For Formulation 2, microparticle yield was 95.7% ± 2.2% and the vaccine encapsulation was 87.0% ± 0.55% as determined by Western blot (Figure 4B). The particle size ranged from 1 to 4 μm with an average size of 3.31 μm. The zeta potential value of these particles was −22.1 ± 1.73 (mV). SEM (Figure 2B) reveals that MPs had irregular shapes. TEM (Figure 3B) indicates that HPV16 VLPs were absorbed into the adjuvant matrix of alhydrogel/MPL.

### 2.2. In Vivo Study—Humoral Immune Response to Transdermal Vaccination

**Figure 5:**
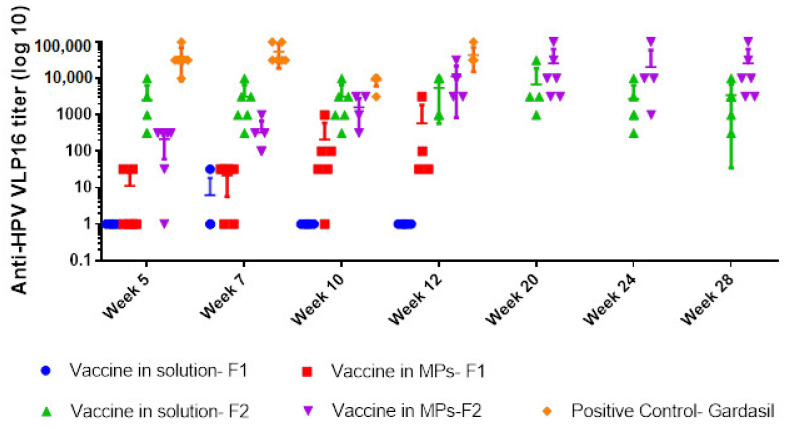
Anti-HPV16 IgG titers (*Y*-axis) in different weeks (*X*-axis) of 5 groups: vaccine in solution and in MPs using Formulation 1 (containing 60% CPD, 15% HPMCAS, 15% EC)—transdermal route, vaccine in solution and in MPs using Formulation 2 (contained 20% CPD, 34% CPD, 34% EC)—transdermal route, and the positive control group (Gardasil)—intramuscular route. The vertical lines show geometric mean titers (log 10) and 95% confidence intervals.

With Formulation 1, one of the six mice was positive for HPV16 IgG in the group treated with the soluble vaccine, compared to four out of six mice in the group treated with vaccine MPs, and all mice (6/6) immunized with Gardasil (positive control group) at week 7. Antibody titers increased from week 5 to week 12 in the MP-treated group but dropped after week 7 in the solution-treated group (Figure 5). Although not statistically significant, antibody titers were higher in the MP-treated group compared to adjuvanted vaccine solution, but lower than titers observed in mice that received Gardasil^®^.

All mice immunized with Formulation 2 in both vaccine solution and vaccine MP groups were positive for HPV16 IgG at week 5. However, both groups had significantly lower titers compared to the positive control group at weeks 5 and 12 (*p* ≤ 0.008). Comparison within the transdermal groups showed an increase in titers with MP vaccination compared to the solution group at week 12. Antibody levels peaked at week 20 for the MP group and were stable until week 28 compared to the solution group (*p* = 0.04), in which antibody levels declined following week 20.

### 2.3. Cell-Mediated Immune Response against HPV16 VLP Antigen

**Figure 6:**
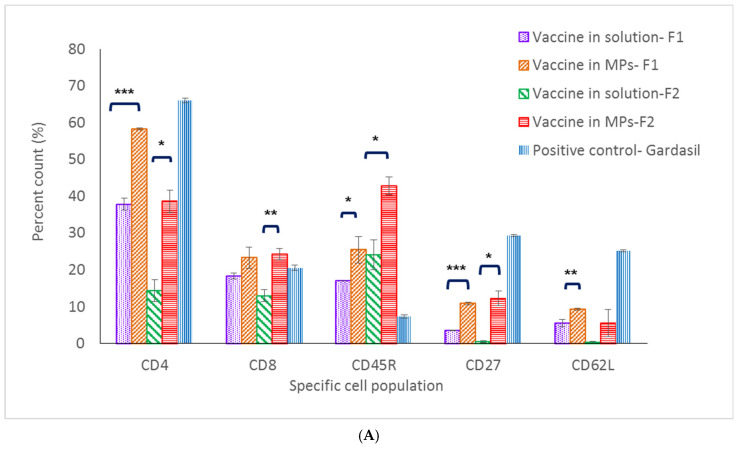

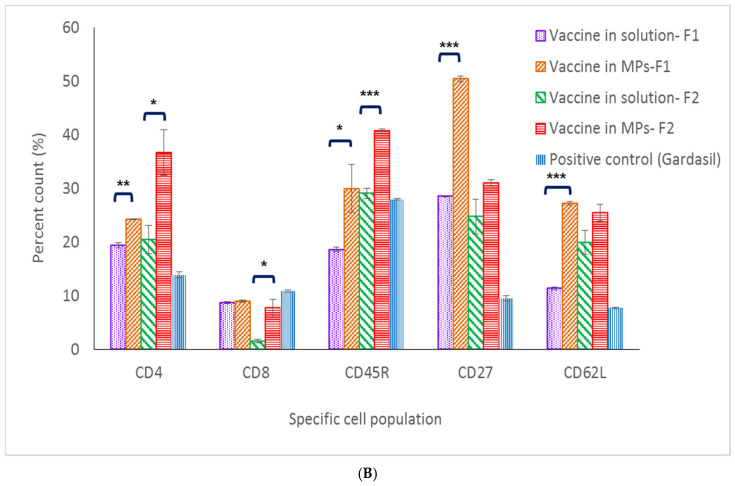
Percent cell count of lymphocyte populations in lymph node (**A**) and the spleen (**B**). (* *p* < 0.5, ** *p* < 0.01, *** *p* < 0.001).

In mice immunized with Formulation 1, except for the CD8^+^ T-cell population, populations (CD4^+^ T cell, memory T and B cells) in the spleen and lymph nodes of the microparticle vaccine group were significantly higher compared to these populations in the vaccine solution group (Figure 6A,B). With Formulation 2, except for the memory B-cell population (CD62L marker), populations in the spleen and lymph nodes of mice immunized with the vaccine MPs were significantly higher compared to populations that received the soluble vaccine (Figure 6A,B).

CD62L, CD27 and CD45R are markers for central memory B- and T-cell populations [20]. In the spleen, memory B- and T-cell populations of mice immunized with the microparticulate vaccine (both Formulations 1 and 2) were significantly higher when compared to the same cell populations in the positive control group. Further, 50.5% and 30.1% percent memory T-cell counts were found in microparticulate vaccine groups (Formulations 1 and 2, respectively), while only a 9.5% cell count was found in the positive control group. In addition, 27.2% and 25.5% memory B-cell counts were found in the microparticulate vaccine groups (Formulations 1 and 2, respectively), whereas only 7.7% of memory B cells were found in the Gardasil group. However, in the lymph nodes, memory B- and T-cell populations in the positive control group were 29.3% and 25.2%, respectively, which were higher than those seen in populations of mice immunized with vaccine microparticles of Formulation 1 (10.5% and 9.3%) and Formulation 2 (12.2% and 5.6%) (Figure 6A,B).

## 3. Discussion

Human papillomavirus vaccines are regarded as the first prophylactic cancer vaccine. They have been proven effective in preventing disease caused by HPV 6, 11, 16, 18, 31, 33, 45, 52 and 58 [20]. However, the current vaccines are expensive to manufacture and administer, thereby making the prospect of alternate vaccines that are efficacious and potentially cheaper more attractive. As a first step, we utilized the advantages offered by microparticle technology combined with needle-free injection to evaluate efficacy of vaccine formulations with HPV16 VLPs.

Formulations 1 and 2 vaccine microparticles consisted of a cellulose-based matrix combining cellulose acetate phthalate (CPD), ethyl cellulose and hypromellose acetate succinate (HPMCAS). CPD and HPMCAS are pH-dependent polymers not soluble under acidic conditions; thus, they are beneficial for a transdermal delivery route, as skin pH is slightly acidic (5.0–6.0) [21]. Formulation 2 was prepared after Formulation 1 with further improvements: (i) reducing CPD in the formulations to increase HPV16 VLP release from the polymer matrix and (ii) absorbing antigen HPV16 VLPs with a different adjuvant combination of MPL and alum prior to spray drying to enhance induction of immune response. However, the absorption of vaccine to the adjuvant combination and reduction of CPD caused lower particle polydispersity and made for a less smooth particle surface. With more than 80% antigen encapsulation, TEM images of Formulation 2 show that most HPV16 VLPs are absorbed to alum + MPL and the VLPs maintained their conformational structure in MPs with polymeric matrices (HPMCAS-CPD-EC) following spray drying, similar to what was observed previously in microparticulate respiratory syncytial virus-like particle vaccines [22].

The transdermal delivery system is being actively researched as a potential route of vaccination due to the presence of Langerhans cells and dermal dendritic cells under the skin that can help mount a robust immune response to vaccination [23]. Both formulations were successful in eliciting HPV16 IgG response when delivered transdermally using AdminPatch^TM^. Though only a few mice in the group immunized with vaccine MP with CT + MPL were seropositive, the antibody titers did increase from week 5 to week 12, which proved MPs maintained the integrity of HPV16 VLPs and the vaccine activated the innate and adaptive immune responses. To improve on these results, changes to concentrations of polymer matrix, adjuvant type and pre-absorption to adjuvant were made for Formulation 2. The altered dosing evaluated with a prime dose of 20 μg HPV16 VLPs/mouse, two times greater than the prime for Formulation 1, increased seropositivity in both solution and MP groups with Formulation 2. We observed higher titers in the solution group with Formulation 2 compared to Formulation 1, indicating that the adsorption of HPV16 VLPs onto MPL and alum may also have enhanced the immunogenicity of the vaccine.

Our results are similar to those published by Corbett et al. [11] and Kines et al. [12] in that the transdermal route produced a robust humoral response. In addition, we showed robust long-term antibody response with microparticulate vaccines (Formulation 2). As expected, antibody titers took longer to peak with the microparticulate vaccine compared to its solution counterpart, but sustained peak levels up to 28 weeks. Encapsulation of HPV16 VLPs in microparticles (1–5 μm) helped to sustain antigen exposure in the body and release HPV16 VLPs slowly from the polymeric matrix.

In the current study, the use of a microparticulate vaccine combined with transdermal delivery did not allow for use of a lower antigen dose. Intramuscular delivery of a lower dose of Gardasil (L1 only VLPs) produced significantly higher titers compared to the transdermal vaccine at the weeks sampled, showing that more improvements to our current formulations are necessary. In a previous study, antibody responses generated against L1 + L2 VLPs were lower compared to L1 VLPs delivered transdermally, but no difference in protection against HPV 16 infection was observed [12]. Characteristics such as differences in purity of VLPs, reduced availability of antigen from adjuvanted spray-dried microparticles and inefficient antigen release could be among possible reasons for higher dosing with current formulations [7].

Along with innate immune response, cell-mediated immune response is also essential in initiating a sustained immune response [24]. CD4^+^ T-cell populations in the spleen and lymph nodes of mice immunized with MPs of both formulations were significantly higher compared to the same populations in the vaccine solution group (including the positive control group) at 32 weeks post-vaccination. These data pinpoint that the MPs induced and extended the helper CD4^+^ T-cell population in secondary lymphoid organs which assist in antibody formation. On the antigen-presenting cells, CD4^+^ T cells recognize peptides presented on MHC class II molecules and play a role in instigating and shaping adaptive immune responses such as Th1/Th2 pathways. CD4^+^ T cells also help the formation process of antibodies from plasma B cells, making the CD4+ T-cell population response essential in vaccination [25]. On the other hand, data on low levels of CD8+ T cells specific against HPV16 VLPs suggest that it might be cleared out from the immune system after 32 weeks. Functions of CD8+ T cells include recognizing peptides presented by MHC class I molecules and helping the immune defense mechanism against intracellular pathogens [26]. Since antigen-presenting cells would engulf either HPV16 VLPs released from MPs or the entirety of vaccine MPs via the phagocytosis pathway, the majority of HPV16 VLPs would be presented on MHC class II, which later communicate with CD4+ T cells in the immune stimulation cascade. Memory B- and T-cell populations are also important for long-term protection against pathogens. Central memory T and B cells proliferate during the secondary expansion or boost doses in vaccination [27]. In this study, markers evaluated for central memory B- and T-cell populations in the vaccine MP group were significantly higher compared to the populations in the vaccine solution group, providing evidence that microparticulate vaccines can stimulate and induce proliferation of central memory B and T cells in the secondary lymphoid system.

## 4. Materials and Methods

### 4.1. Chemicals

Common chemicals of aqueous dispersion (Aquacoat^®^ CPD), hydroxyl propyl methylcellulose acetate succinate (HPMCAS, AQOAT) and ethyl cellulose 30% (*w*/*v*) aqueous dispersion (AQOAT) were obtained from FMC biopolymers (Philadelphia, PA, USA). Trehalose was purchased from Sigma-Aldrich (St. Louis, MO, USA). Aleuria aurantia lectin (AAL) was purchased from Vector Laboratories (Burlingame, CA, USA). Alhydrogel^®^ adjuvant 2% (alum), cholera toxin (B subunit) (CT) and monophosphoryl lipid A (MPL) were purchased from InvivoGen (San Diego, CA, USA). All other analytical-grade chemicals used in this study were purchased from Fisher Scientific (Norcross, GA, USA).

Eight-week old (20–25 g) female BALB/c mice were (Charles-River Laboratories, Wilmington, MA, USA) quarantined for one week prior to start of the study. All mice were housed and provided with water and food in an animal facility with a 12 h light and dark cycle and at 71 °F.

### 4.2. Preparation and Quantification of HPV16 Virus-like Particles (VLP)

HPV16 L1 + L2 VLPs were produced using plasmid p16SheLL transfected into HEK 293 TT cells (Received from John T. Schiller, NCI, NIH) [28]. The crude VLP stocks were recovered by cell lysis after 48 h of incubation and purified by ultracentrifugation using Optiprep^TM^ (Sigma-Aldrich, St. Louis, MO, USA) gradient in Dulbecco’s Phosphate Buffered Saline (D-PBS) (pH 7.4) plus 0.5 M NaCl [29]. Optiprep^TM^ and NaCl were removed from the collected fractions by a diafiltration process using D-PBS (pH 7.4). The fractions were stored at −80 °C. Total protein content was quantified using a Coomasie Plus (Bradford) Protein Assay kit (Thermo Scientific, Waltham, MA, USA). The integrity/quality of the VLP fractions was evaluated using type-specific monoclonal antibodies that recognize a conformational epitope on the intact VLP [30]. VLP fractions with reactivity equal to or higher than the pre-qualified VLP used as a laboratory reference were pooled to make VLP stock used for formulation. Pooled VLPs were stored at −80 °C until use. VLP conformation was visualized using TEM.

### 4.3. Preparation of Vaccine Microparticles (MP)

The microparticles of HPV16 VLPs were prepared using a Buchi B-290 spray dryer. For Formulation 1 (F1), the microparticles contained 60% (*w*/*w*) of CPD, 15% (*w*/*w*) of ethyl cellulose, 15% (*w*/*w*) of hydroxyl propyl methylcellulose acetate succinate (HMCAS; FMC biopolymers; Philadelphia, PA, USA), 4.74% (*w*/*w*) of trehalose (Sigma-Aldrich, St. Louis, MO, USA), 4% (*w*/*w*) of chitosan and 1% (*w*/*w*) HPV16 VLP. Briefly, CPD dispersion (30% *w*/*v*) and HPMCAS were diluted separately in deionized water at a concentration of 5 mg/mL with stirring. Since both CPD and HPMCAS are pH-dependent polymers, 1 N NaOH was used to adjust the pH of CPD dispersion and HPMCAS to pH 6.0 and 8.0, respectively. Ethyl cellulose (EC) was added next, with the ratio of CPD: HPMCAS: EC as 4:1:1. An amount of 4% (*w*/*w*) chitosan glycol was added to the solution, followed by 0.25% (*w*/*w*) of Aleuria aurantia lectin (AAL, Vector Laboratories, Burlingame, CA, USA) and 0.01% (*w*/*w*) Tween-20. The pH of the solution was adjusted to 7.0–7.4, following which, trehalose and HPV16 VLPs were added into the matrix prior to spray drying. The final solution was then constantly stirred at 50 rpm during the spraying process to maintain homogeneity. Adjuvant micro/nanoparticles with monophosphoryl lipid A (MPL; InvivoGen, San Diego, CA, USA) and cholera toxin B subunit (CT; InvivoGen) were made separately using the same procedure as HPV16 VLPs microparticles, in which 1% (*w*/*w*) antigen in the formulation was replaced with 1% (*w*/*w*) adjuvants (MPL and CT).

For Formulation 2 (F2), the HPV16 VLPs were formed as an enteric-coated polymer matrix or microparticle. This matrix consisted of 20% (*w*/*w*) of CPD, 34% (*w*/*w*) of ethyl cellulose, 34% (*w*/*w*) of hypromellose acetate succinate (HAS) (Sigma-Aldrich, St. Louis, MO, USA), 4.74% (*w*/*w*) of trehalose, 4% (*w*/*w*) of chitosan and 5% (*w*/*w*) of combined HPV16 VLP, MPL and alum with a mass ratio of 1 µg: 2.5 µg: 5 µg. The polymeric matrix was prepared similar to Formulation 1, except that HPV16 VLPs were mixed with MPL and alum 4 h prior to the spray-drying process to maximize the adsorption of antigen into adjuvants.

The Buchi B290 spray dryer was set with inlet temperature at 120 °C with the inlet flowrate at 3.0 mL/min. Since the protein-based vaccine (HVP16 VLP) was sprayed together with the matrix, the cyclone powder collector was covered by a cooling jacket connected to a condenser at 0 °C. The outlet temperature was maintained at 70 °C throughout the process. The extent of reduction of pressure across the cyclone and filter of the spray dryer was measured inside the dryer’s aspirator settings between 50 and 100%. Then, the dry particulate vaccine was collected in glass vials and stored at −20 °C for future uses.

### 4.4. Physical Characterization of Microparticles (MP)

The size of the particles was determined using the Spectrex Laser Particle Counter (Spectrex, Redwood City, CA, USA). One-milligram particles were suspended in 1 mL citric acid buffer (10 mM, pH 4.0), vortexed, and read on the particle counter. The size was measured in triplicate for adjuvant- and antigen-loaded particles.

Three-milligram microparticles were suspended in 3 mL of citric acid buffer (100 mM, pH 4.0), transferred to a zeta potential measurement cuvette and measured using a Malvern Zetasizer (Malvern Instruments Ltd., Worcestershire, UK). Scanning electron microscopy (SEM) was performed to evaluate size distribution and surface morphology. Microparticles were placed onto metal stubs using carbonate double-sided tapes. Images were captured using Phenom Pure Desktop with ×7500 (view angle was 28.7) and an accelerating voltage of 20 kV.

### 4.5. Western Blot Analysis for HPV16 VLP in Microparticles

For this analysis, 10% Tris.HCl gels (Biorad, Hercules, CA, USA) were used for gel electrophoresis following the manufacturer’s protocol. All blue precision standard (Biorad) was used as the protein standard. HPV16 VLPs were released from microparticles by resuspension in phosphate-buffered saline (PBS). Different dilutions of the resuspended vaccine particle preparations, along with a standard curve of known amounts of HPV16 VLPs spiked into blank microsphere suspension, were run on the gel. The gel was transferred onto a nitrocellulose membrane using the i-blot system (Thermo Scientific, Waltham, MA, USA). L1 protein was detected using Camvir-1 (Abcam, Cambridge, MA, USA) followed by FITC-labeled goat anti-mouse antibody. The membrane was scanned on the Typhoon imager (GE healthcare, Pittsburgh, PA, USA) and quantitative analysis of HPV16 L1 within the microparticles was conducted using ImageJ software (open-source program).

### 4.6. Transmission Electron Microscopy

HPV16 VLPs were released from microparticles by resuspension in PBS. Samples were placed on Formvar/carbon-coated 300-mesh nickel grids (pretreated with 1% Alcian Blue) and stained with 5% ammonium molybdate, pH 6.9 and 1% trehalose. Stained grids are viewed on FEI Tecnai Bio-twin at 120 kV. Grids were scanned. Adequately stained and average-looking grid squares (with respect to the rest of the grids squares present) were selected for further inspection and image capture.

### 4.7. In Vivo Study in BALB/c Mice

The animal study was approved by the University Institutional Animal Care and Use Committee (IACUC) with the project number A1312017.65. Three groups (six mice per group) of 8- to 10-week-old female BALB/c mice were used to evaluate the immune response induced by HPV16 VLPs and the adjuvant combination of MPL and CT. Mice in Groups 1 and 2 were immunized via the transdermal route with vaccine/adjuvant in solution and in MPs, respectively. Mice in Group 3 were immunized intramuscularly with a commercial HPV vaccine (Gardasil) and served as the positive control group. Mice in Groups 1 and 2 received 10 μg of HPV16 VLPs, 20 μg of MPL and 40 μg of CT (approximately 1.2 mg of vaccine MPs, 2.4 mg MPL MPs and 4.8 mg CT MPs) per dose at weeks 0, 2, 4 and 8. The adjuvant/vaccine in solution and MPs were suspended in a total of 200 μL of 100 mM citric acid buffer (pH 4.0) for dosing. Vaccine MPs and adjuvant MPs were weighed and transferred into a 1.7 mL centrifuge vial before being suspended in citrate buffer for dosing. Mice in Group 3 received three doses at week 0, 2 and 4; each dose contained 3 µg HPV 16 VLPs, 3 µg HPV18 VLPs, 1.5µg HPV6 VLPs, 1.5 µg HPV11 VLPs and 13.3 µg amorphous aluminum hydroxyphosphate sulfate adjuvant. Blood samples were obtained from the facial vein of mice at week 0 (pre-dosing), 5, 7, 10 and 12. Blood samples were collected in sterile polypropylene tubes and centrifuged at 5000 rpm for 10 min. Serum samples were collected and stored at −80 °C for detecting antibodies against HPV16.

The study to evaluate Formulation 2 was also conducted in two groups: HPV16 VLPs and a combination of alhydrogel^®^/MPL in solution and in MPs via the transdermal route. The testing was performed similar to Formulation 1, except for the dosing. The prime dose was administered at week 0 which contained 20 μg of HPV16 VLPs, 50 μg of MPL and 100 μg of alum in about 3.5 mg of MPs. Whereas the booster doses consisted of half of the amount of VLPs (10 μg), MPL (25 μg) and alum (50 μg), in comparison with the prime dose, and were administered at weeks 2, 4 and 8. Blood samples were obtained from the facial vein of mice at weeks 0 (pre-dosing), 5, 7, 10, 12, 20, 24 and 28, and were processed as above.

### 4.8. HPV16 VLP-Based IgG ELISA

ELISA was conducted using serum samples which were thawed on ice before testing. Sera from mice at week 0 (pre-dosing) and pooled sera from the group inoculated with Gardasil^®^ served as negative and positive controls, respectively. HPV16 VLP diluted to 0.5 µg/mL in PBS was used as an antigen to coat microtiter plates and incubated at 4 °C overnight. Following each incubation step, the plates were washed four times with PBST (PBS–0.1% Tween-20) using an automated plate washer (ELx 405, Biotek-Agilent, Santa Clara, CA, USA). Plates were blocked for one hour with solution that contained TBST (10 mM Tris.HCl pH 8.0, 0.15 M NaCl and 0.5% Tween-20, along with 10% goat serum (Thermo Fisher, Waltham, MA, USA) and 50% SuperBlock [Pierce]) at room temperature (24 °C ± 2) on a plate rotator set at 60 rpm. Both the control and test sera were diluted three-fold starting at 1:31.6, for a total of 6–10 dilutions in sera diluent (TBST with 10% goat serum, 10% Super-Block). An amount of 50 µL of diluted sample was added to the washed plate followed by incubation at 37 °C for one hour in the plate shaker incubator (Millennium 2000, Jencons Scientific, Leighton Buzzard, England, UK). This was followed by adding 100 µL of 1:2000 dilution of anti-mouse IgG conjugated to alkaline phosphatase diluted in conjugate diluent (in TBST with 10% goat serum, 10% Super-Block) and incubated for two hours at 37 °C. The substrate solution (2 mg/mL) was prepared by dissolving alkaline phosphatase substrate tablets (Sigma, St. Louis, MO, USA) in sodium bicarbonate buffer (0.1 M NaHCO_3_, 0.01 M MgCl_2_, pH 9.5 (Sigma, St. Louis, MO, USA). Then, 50 µL of substrate was added to each well. The plates were incubated at room temperature (24 °C ± 2) on a rotator at 60 rpm. After 45 min of rotation, absorbance was determined at 405 nm using a plate reader (Wallac-Victor2^TM^; Perkin Elmer, Waltham, MA, USA). The threshold for seropositivity was established as average OD plus 3 standard deviations of results observed in sera from mice at week 0. In each group, the highest dilution of sera with OD value above threshold was considered as the final positive titer. The final antibody titers were averaged to calculate the geometric mean titer and compared between weeks for each group.

### 4.9. Flow Cytometry Cell Sorting (FACS) Analysis

At week 40, all groups of mice from both studies were euthanized to collect lymphatic organs such as the spleen and lymph nodes. Then, the cell suspensions of these secondary lymphoid organs were labeled with anti-mouse CD4 PE, anti-mouse CD8a FITC, anti-mouse CD45R (B220) FITC, anti-mouse CD62L APC and anti-mouse CD27 APC (eBioscience). The procedure was conducted according to the manufacturer’s protocol [31]. The labeled cells were then analyzed by flow cytometric analysis using a BD Accuri^®^ C6 flow cytometer.

### 4.10. Statistics

Data were analyzed by a multiple *t*-test model with pair-wise comparisons using the Holm–Sidak method. All statistical tests were performed using GraphPad Prism v7.0 (La Jolla, CA, USA). For each test, a *p*-value less than 0.05 was considered significant.

## 5. Conclusions

We successfully developed a formulation for micro/nanoparticulate vaccines for transdermal delivery systems that induced a robust immune response to HPV16 VLPs. Both formulations consisted of cellulose CPD, HPMCAS and EC as a polymeric matrix incorporated with HPV16 VLPs. The physical properties of HPV16 vaccines and the integrity of the antigens were characterized and were found to be suitable for transdermal vaccination. Formulation 1 proved the significance of micro/nanoparticle technology in vaccine development, as the particulate vaccine elicited better humoral immune response (HPV16 IgG titer). However, Formulation 2, with a reduction of cellulose acetate phthalate (from 60% to 20%) and the adsorption of HPV16 VLPs into alum and MPL, helped to induce higher HPV16 IgG responses and sustain a long-term immune response. The cellular immune response (CD4^+^ T cells, memory B- and T-cell populations in the spleen and lymph nodes) were also elevated with MPs as compared to the vaccine solution. Further improvements to the formulations that utilize lower antigen doses, alternative microneedle patches and stability of microparticulate vaccines over time and at different temperatures should be evaluated in the future.

## Data Availability

Data is contained within the article.
